# Refeeding in crisis settings: Implications on health care needs in Gaza

**DOI:** 10.1371/journal.pgph.0003280

**Published:** 2024-05-24

**Authors:** Pooja Yerramilli

**Affiliations:** 1 Department of Medicine, George Washington University School of Medicine and Health Sciences, Washington, District of Columbia, United States of America; 2 Johns Hopkins Center for Humanitarian Health, Johns Hopkins University, Baltimore, Maryland, United States of America; PLOS: Public Library of Science, UNITED STATES

For months, humanitarian organizations and international agencies have sounded alarms over the imminent famine in Gaza. The Integrated Food Security Phase Classification (IPC) Report in March 2024 projected that, in the absence of cessation of hostilities and sustained access to essential aid and services including food, 1.1 million people–amounting to half the population in Gaza–will experience catastrophic food insecurity between mid-March and mid-July. Indeed, particularly in Northern Gaza, starvation has significantly increased, with rates of acute malnutrition rising from between 1% and 6.8% in January 2024 to between 12.5% and 16.5% the next month [1].

The rise of widespread malnutrition raises significant concerns regarding the associated short, medium and long-term health consequences that will arise in the context of a collapsing health system in Gaza. As a direct consequence of severe acute malnutrition, children can experience wasting and death in the immediate term—from, for example, hypoglycemia, hypothermia, infection, dehydration, and anemia—and stunting over time [2]. Malnourished children and adults are more vulnerable to severe illness of all causes [3]; in Gaza, this is exacerbated by the lack of access to water, sanitation and hygiene which has facilitated the spread of infectious disease such as Hepatitis A [1]. Beyond these health impacts, evidence suggests that the consequences of malnutrition among children are life-long; lasting systemic inflammation may increase risks of cardiovascular and metabolic diseases in adulthood [4].

What has been less well-described, however, is the monitoring that is required when malnourished individuals are re-fed, and the medical support that will be needed alongside attempts to restore food access in Gaza. Depending on the level of acute malnutrition a young child experiences, they require consistent community-based treatment at the least–including delivery of nutritional supplements, specialized nutritious foods specifically formulated to treat malnutrition, and possibly oral antibiotics–and hospital-based treatment for severe cases that require medical stabilization and more intensive care including IV medications [2]. But even in older children and adults, healthcare needs to treat malnutrition can be significant. Namely, in the first one to two weeks that malnourished patients are fed, they are at heightened risk of electrolyte and fluid shifts that can result in severe medical complications including seizures, respiratory failure, cardiac arrest and death–otherwise known as refeeding syndrome [5].

In countries such as the United States, individuals at high risk of re-feeding syndrome are typically observed carefully in medical facilities. These include patients who weigh less than 70% of ideal body weight or who have rapidly lost large amounts of weight due to conditions such as anorexia nervosa, alcohol use disorder, cancer, and other critical illnesses. Refeeding syndrome can be avoided, particularly for patients at highest risk, through daily observation of clinical status and biomedical testing, alongside methodical titration of electrolyte repletion and nutritional intake to prevent complications [5].

While more is known about treatment of malnutrition in young children, little has been published explicitly about the incidence of complications like refeeding syndrome among older children and adults in crisis settings; however our historical understanding of and current clinical guidelines for treatment of malnutrition across all ages suggest that access to food—including specialized nutritious foods, which have their own complex supply chain considerations [6]—must be matched by commensurate access to health services in these contexts. Anecdotal accounts of refeeding syndrome trace back to World War II, including among released prisoners of war [7]. In recent months, news reports have suggested that some of the released Israeli hostages were medically monitored in hospitals for, among other conditions, refeeding syndrome, given concerns and uncertainty regarding their access to food while in captivity [8].

These insights and practices suggest that the impacts of malnutrition in Gaza will not be easily or quickly undone with a focus on food aid alone. Based on the rising rates of acute malnutrition in Gaza, a high percentage of the population is likely at risk for immediate health consequences that food consumption cannot solve in the absence of the health systems needed to monitor and protect against complications. Between October 2023 and end of January 2024, 84% of health facilities in Gaza were destroyed or damaged, with the remaining facilities challenged by lack of electricity and water [9]. As of March 2024, there were four hospitals and two clinics remaining in Gaza Governorate and two hospitals and two clinics remaining in North Gaza, reported to be functioning at low levels [1].

The health sector needs in the context and aftermath of the protracted crisis in Gaza are tremendous. Recent projections suggest that in the absence of ceasefire, excess mortality between February and August 2024 will exceed 58,000; but even in a ceasefire scenario, excess mortality is projected to reach over 6,500 over this same period–from a combination of, for example, trauma related injuries, infectious disease, non-communicable diseases, and maternal and neonatal conditions, all of which are exacerbated by malnutrition [3]. It is clear that what remains of the health system in Gaza will be unable to cope with these rising disease burdens.

That there remain uncertainties regarding the incidence, monitoring, and treatment of the health consequences of mass malnutrition in conflict zones, including the risk of refeeding syndrome, suggests that more attention and knowledge sharing is needed on this subject. But in the immediate term, clinical evidence and protocols on malnutrition argue that the current crisis cannot be mitigated through ad hoc provisions of food aid [2, 5, 10]. Efforts to restore access to food must be met with immediate, coordinated, and commensurate attention on access to health services to both monitor and treat people with malnutrition, its health complications, and other causes of illness; but, of course, this will remain infeasible in the context of ongoing and escalating attacks, blockade, destruction of health infrastructure, and restricted and unprotected movement of health and humanitarian workers in Gaza.
